# Systemic Anti‐Inflammatory Effects of Isotretinoin: Evaluation of Red Cell Distribution Width to Lymphocyte and Platelet Ratios as New Hematological Markers and Clinical Outcomes in Acne Vulgaris

**DOI:** 10.1111/jocd.70108

**Published:** 2025-03-10

**Authors:** Abdullah Demirbas, Gozde Ulutaş Demirbas, Esin Diremsizoglu, Gizem Islamoglu

**Affiliations:** ^1^ Department of Dermatology Kocaeli University Faculty of Medicine Kocaeli Turkiye; ^2^ Department of Dermatology Kocaeli City Hospital Kocaeli Turkiye; ^3^ Private Clinic Konya Turkiye

**Keywords:** acne vulgaris, inflammation markers, Isotretinoin, neutrophil‐lymphocyte ratio (NLR), red cell distribution width (RDW)

## Abstract

**Background:**

Acne vulgaris is a chronic inflammatory skin disease influenced by systemic immune responses. While isotretinoin is the most effective treatment for severe acne, its effects on hematological markers of inflammation, in particular red cell distribution width (RDW)‐to‐lymphocyte and RDW‐to‐platelet ratios, remain unclear.

**Methods:**

This retrospective study analyzed 450 acne patients treated with isotretinoin for at least 24 weeks. Hematological parameters, including complete blood count (CBC) indices, RDW‐derived ratios, neutrophil‐to‐lymphocyte ratio (NLR), and monocyte‐to‐lymphocyte ratio (MLR), as well as biochemical markers (ALT, AST, ALP) and clinical measures (lesion counts, Global Acne Grading Score [GAGS], Dermatology Life Quality Index [DLQI], SF‐36, and Hospital Anxiety and Depression Scale [HADS]) at baseline and weeks 8, 16, and 24.

**Results:**

Isotretinoin treatment was associated with significant reductions in systemic inflammatory markers, including WBC count, NLR, and MLR, while lymphocyte count increased (*p* < 0.001). The ratios of RDW to lymphocytes and RDW to platelets also decreased over time (*p* < 0.001). Liver enzymes remained stable. Clinical assessments showed significant improvements in acne severity, quality of life, and mental health scores (*p* < 0.001). Adverse events were reported in 90.7% of patients, with musculoskeletal symptoms and constipation more common than previously reported.

**Conclusion:**

This study highlights the systemic immunomodulatory effects of isotretinoin, particularly the significant changes in RDW‐derived ratios, suggesting their potential as novel inflammatory markers. These findings reinforce the broader effects of isotretinoin beyond sebaceous gland suppression and support its role in the regulation of systemic inflammation.

## 1 Introduction

Acne vulgaris is a chronic inflammatory skin disease of the pilosebaceous unit with a prevalence ranging from 85% in adolescents to persistent cases in adults. Although the pathogenesis of acne is multifactorial, inflammation plays a crucial role in its development and progression. Dysregulation of the immune response, increased sebum production, proliferation of *Propionibacterium acnes* , and altered keratinization all contribute to persistent inflammation, leading to comedones, papules, pustules, and nodules [1].

Isotretinoin (ISO), a systemic retinoid, is the most effective treatment for moderate to severe acne vulgaris, particularly in cases resistant to conventional therapies. It reduces sebaceous gland activity, normalizes keratinocyte differentiation, and exerts anti‐inflammatory properties. However, the exact mechanisms underlying its effects on systemic inflammatory markers remain unclear [2, 3, 4].

Several hematological indices, such as neutrophil‐to‐lymphocyte ratio (NLR), monocyte‐to‐lymphocyte ratio (MLR), red cell distribution width (RDW), and platelet‐related markers, have been proposed as potential indicators of systemic inflammation. These markers have been studied in autoimmune, cardiovascular, and chronic inflammatory diseases, and their role in acne vulgaris and response to isotretinoin treatment continues to be investigated [4, 5, 6, 7, 8, 9, 10, 11, 12, 13, 14, 15, 16].

This study aims to evaluate the systemic inflammatory effects of isotretinoin treatment, focusing on RDW/lymphocyte and RDW/platelet ratios as potential novel inflammatory markers. In addition to changes in WBC, neutrophil, lymphocyte, monocyte, RDW, platelet indices, and other inflammatory ratios (NLR, MLR), we will also assess changes in liver enzymes (ALT, AST, ALP), clinical acne severity (lesion counts, Global Acne Grading Score [GAGS]), and quality of life parameters (DLQI, SF‐36, HADS) throughout treatment. These results may contribute to a better understanding of the systemic immunomodulatory effects of isotretinoin and the implications for patient monitoring.

## 2 Materials and Methods

This retrospective study included 450 patients diagnosed with acne vulgaris and treated with oral isotretinoin for at least 24 weeks. Patients were selected from those attending routine dermatological follow‐up appointments. The study followed the Declaration of Helsinki, and ethical approval was obtained from the institutional review board.

Patients aged 16 to 45 years with complete clinical and laboratory records were included. Exclusion criteria were active infections, anemia, hematological or autoimmune diseases, chronic inflammatory conditions, and the use of medications that could affect hematological or biochemical parameters, including systemic steroids, immunosuppressants, and lipid‐lowering agents. Pregnant or lactating women and patients with incomplete follow‐up data were also excluded.

Isotretinoin treatment was administered at a daily dose of 0.5–1 mg/kg, with dose adjustments based on tolerability and adverse events. Laboratory and clinical assessments were performed at baseline and weeks 8, 16, and 24 of treatment.

Data collection included demographic characteristics (age, sex, BMI, age of acne onset, and duration of acne), hematological parameters (WBC, neutrophil, lymphocyte, monocyte, platelet count, RDW), inflammatory markers (NLR, MLR, RDW‐to‐lymphocyte ratio, and RDW‐to‐platelet ratio), and biochemical markers (ALT, AST, ALP). Clinical assessments included lesion counts (comedones, papules, and pustules), GAGS, Dermatology Life Quality Index (DLQI), Hospital Anxiety and Depression Scale (HADS), and SF‐36 physical and mental health scores. Treatment adherence and adverse events were recorded at each visit.

## 3 Statistical Analysis

All statistical analyses were performed using SPSS version 26.0 (SPSS Inc., Chicago, IL, USA). The normality of data distribution was assessed using the Shapiro–Wilk test. As the variables did not follow a normal distribution, non‐parametric tests were used. Changes in continuous variables over time were analyzed using the Friedman test and, if a significant difference was found, pairwise comparisons were made using the Wilcoxon signed‐rank test. Categorical variables were presented as frequencies and percentages and analyzed using the chi‐squared test. Continuous variables were expressed as medians and interquartile ranges (IQRs). A *p* < 0.05 was considered statistically significant.

## 4 Ethical Approval

All procedures adhered to the tenets of the Declaration of Helsinki and were approved by an Institutional Review Board (Decision date and number: 2020/499).

## 5 Results

The study included 450 patients, 223 males (49.6%) and 227 females (50.4%). The median age was 29.00 years (IQR: 22.75–34.00). The median BMI was 26.30 kg/m^2^ (IQR: 22.40–30.33). The median age of onset of acne was 16.00 years (IQR: 13.00–20.00), and the median duration of acne was 9.00 years (IQR: 7.00–14.00) (Table 1).

**TABLE 1:** Descriptive characteristics and adverse events of the study population.

Variable	*N* (%)/median (IQR)
Male	223 (49.6%)
Female	227 (50.4%)
Age (years)	29.00 (22.75–34.00)
BMI (kg/m^2^)	26.30 (22.40–30.33)
Acne onset age (years)	16.00 (13.00–20.00)
Acne duration (years)	9.00 (7.00–14.00)
No adverse events	42 (9.3%)
Reported adverse Events	408 (90.7%)
Back pain	60 (13.3%)
Skin dryness	57 (12.7%)
Muscle pain	56 (12.4%)
Constipation	54 (12.0%)
Hair loss	50 (11.1%)
Eye dryness	46 (10.2%)
Lip dryness	43 (9.6%)
Nosebleeds	42 (9.3%)

Abbreviation: BMI, body mass index.

Hematological, biochemical, and clinical parameters were assessed at baseline and weeks 8, 16, and 24. A significant decrease in WBC count was observed from baseline (6.96 [5.48–8.46] × 10^9^/L) to week 24 (5.57 [4.38–6.77] × 10^9^/L, *p* < 0.001). Similarly, the neutrophil count showed a progressive decrease from 4.16 [3.17–4.98] × 10^9^/L at baseline to 3.10 [2.38–3.73] × 10^9^/L at week 24 (*p* < 0.001), whereas the lymphocyte count increased over time (1.99 [1.55–2.56] × 10^9^/L to 2.30 [1.71–2.82] × 10^9^/L, *p* < 0.001).

**FIGURE 1:**
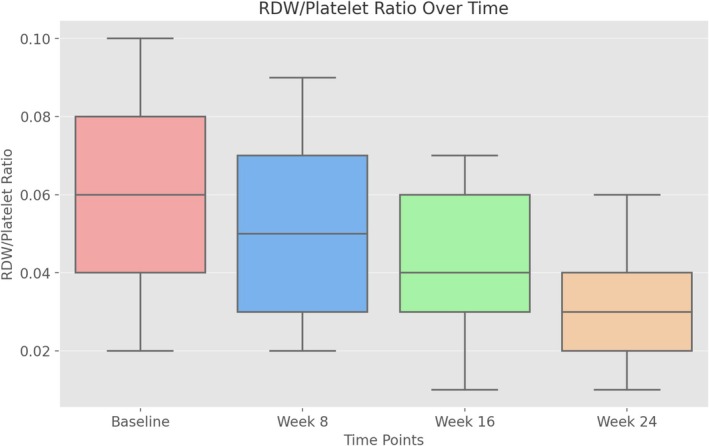
Decline in RDW/lymphocyte ratio over time. The RDW/lymphocyte ratio decreased progressively from baseline to week 24.

**FIGURE 2:**
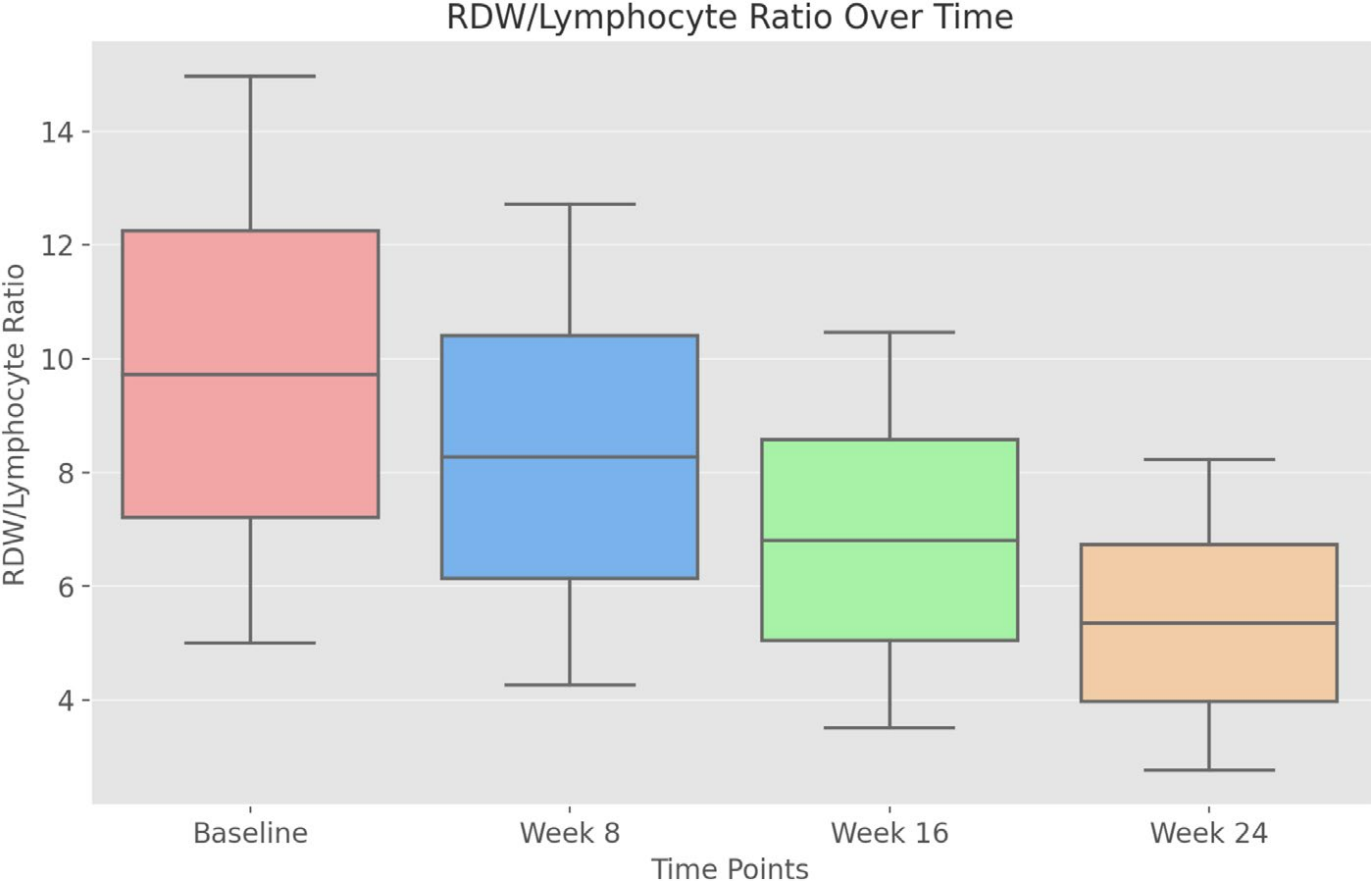
Decline in RDW/platelet ratio over time. The RDW/platelet ratio showed a consistent decline from baseline to week 24.

Inflammatory ratios, including neutrophil to lymphocyte ratio (NLR) and monocyte to lymphocyte ratio (MLR), decreased significantly over time (*p* < 0.001). The RDW to lymphocyte ratio and the RDW to platelet ratio also showed a significant decrease (*p* < 0.001) (Table 2, Figures 1 and 2).

**TABLE 2:** Hematological, biochemical outcomes over time.

Variable	Baseline median, IQR	Week 8 median, IQR	Week 16 median, IQR	Week 24 median, IQR	*p*
WBC (×10^9^/L)	6.96 (5.48–8.46)	6.26 (4.93–7.61)	5.91 (4.66–7.19)	5.57 (4.38–6.77)	< 0.001
Neutrophil (×10^9^/L)	4.16 (3.17–4.98)	3.54 (2.69–4.23)	3.33 (2.54–3.98)	3.10 (2.38–3.73)	< 0.001
Lymphocyte (×10^9^/L)	1.99 (1.55–2.56)	2.10 (1.63–2.69)	2.16 (1.67–2.77)	2.30 (1.71–2.82)	< 0.001
Monocyte (×10^9^/L)	0.40 (0.27–0.56)	0.36 (0.24–0.50)	0.34 (0.23–0.48)	0.33 (0.22–0.45)	< 0.001
RDW (%)	13.85 (12.62–14.50)	13.57 (12.37–14.21)	13.30 (12.12–13.92)	13.02 (11.85–13.63)	< 0.001
Platelet (×10^3^/μL)	313.79 (231.30–381.54)	276.14 (203.54–335.76)	266.73 (196.60–324.31)	251.04 (185.04–305.23)	< 0.001
RDW/lymphocyte ratio	6.69 (5.29–8.75)	6.25 (4.93–8.16)	5.95 (4.70–7.76)	5.72 (4.51–7.47)	< 0.001
RDW/platelet ratio	0.0432 (0.0357–0.0579)	0.0481 (0.0398–0.0644)	0.0488 (0.0404–0.0654)	0.0507 (0.0420–0.0680)	< 0.001
Neutrophil/lymphocyte ratio (NLR)	2.02 (1.55–2.60)	1.63 (1.26–2.10)	1.49 (1.15–1.92)	1.38 (1.06–1.77)	< 0.001
Monocyte/lymphocyte ratio (MLR)	0.20 (0.14–0.27)	0.17 (0.12–0.23)	0.16 (0.11–0.21)	0.15 (0.10–0.20)	< 0.001
ALT (U/L)	15.68 (12.78–19.02)	15.46 (12.80–18.78)	15.93 (12.71–18.88)	16.23 (13.31–18.84)	0.224
AST (U/L)	13.85 (11.81–15.84)	14.10 (12.01–16.11)	14.00 (12.03–15.94)	13.80 (12.03–16.03)	0.428
ALP (U/L)	45.71 (37.44–52.71)	45.56 (37.77–52.92)	44.85 (37.29–52.87)	45.62 (36.91–51.79)	0.865

Abbreviations: ALT, alanine aminotransferase; AST, aspartate aminotransferase; ALP, alkaline phosphatase; MLR, monocyte‐to‐lymphocyte ratio; NLR, neutrophil‐to‐lymphocyte ratio; RDW, red cell distribution width; WBC, white blood cell count.

Regarding liver function tests, ALT, AST, and ALP levels did not show significant changes over time (*p* = 0.224, *p* = 0.428, *p* = 0.865).

**FIGURE 3:**
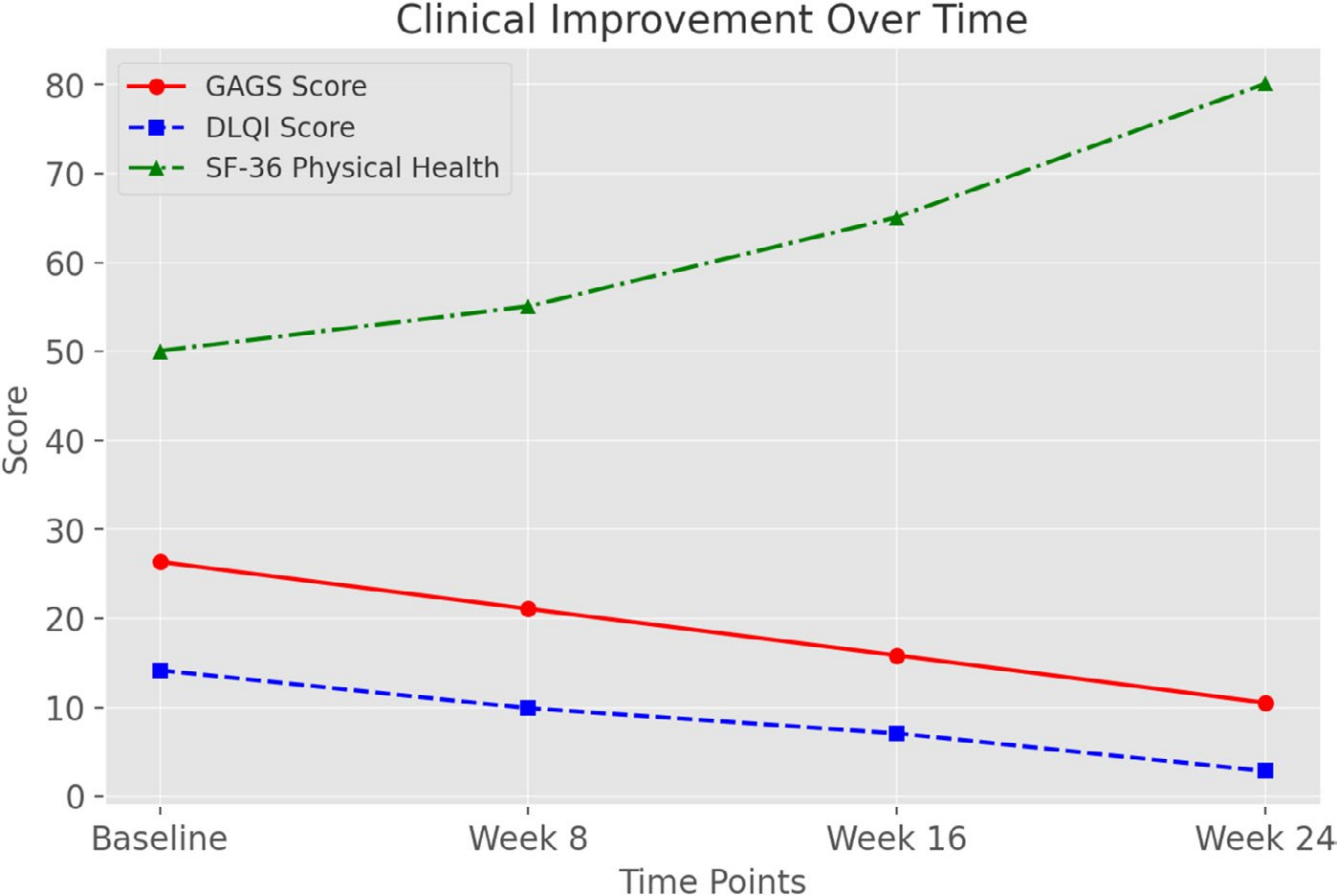
Clinical improvement over time. GAGS and DLQI scores decreased over time, showing improvement in acne severity and quality of life. The SF‐36 physical health score increased, indicating better physical well‐being during isotretinoin treatment.

Clinically, there was a significant improvement in lesion counts (comedones, papules, and pustules) and GAGS scores, which progressively decreased (*p* < 0.001 for all). DLQI scores improved from 14.00 [6.00–22.00] at baseline to 2.80 [1.20–4.40] at week 24 (*p* < 0.001), reflecting improved quality of life. Similarly, SF‐36 physical and mental health scores improved significantly (*p* < 0.001), while HADS scores decreased (*p* < 0.001), indicating reduced symptoms of anxiety and depression (Table 3, Figure 3).

**TABLE 3:** Clinical and quality of life outcomes over time.

Variable	Baseline median, IQR	Week 8 median, IQR	Week 16 median, IQR	Week 24 median, IQR	*p*
Lesion count (comedone)	35.00 (27.00–43.00)	28.00 (22.00–34.00)	21.00 (16.00–26.00)	14.00 (11.00–17.00)	< 0.001
Lesion count (papule)	19.00 (14.00–24.00)	15.00 (11.00–19.00)	11.00 (8.00–14.00)	8.00 (6.00–10.00)	< 0.001
Lesion count (pustule)	12.00 (8.00–16.00)	10.00 (6.00–13.00)	7.00 (5.00–10.00)	5.00 (3.00–6.00)	< 0.001
SF‐36 (physical health)	52.00 (27.00–72.00)	57.20 (29.70–79.20)	67.60 (35.10–93.60)	83.20 (43.20–115.20)	< 0.001
SF‐36 (mental health)	48.00 (21.00–75.00)	52.80 (23.10–82.50)	67.20 (29.40–105.00)	86.40 (37.80–135.00)	< 0.001
HADS	20.00 (11.00–31.00)	17.00 (9.35–26.35)	12.00 (6.60–18.60)	6.00 (3.30–9.30)	< 0.001
Treatment adherence (%)	75.00 (63.00–86.00)	75.00 (62.00–87.00)	74.00 (61.00–86.00)	74.00 (61.00–87.00)	0.991
First improvement time (weeks)	11.00 (7.00–15.00)	11.00 (7.75–16.00)	11.50 (7.75–15.00)	11.00 (8.00–15.00)	0.543

Abbreviations: DLQI, dermatology life quality index; GAGS, global acne grading score; HADS, hospital anxiety and depression scale; SF‐36, short form‐36 health survey.

Treatment adherence remained stable over time (*p* = 0.991), and the time to first improvement did not differ significantly between follow‐ups (*p* = 0.543).

Out of 450 patients, a total of 408 patients (90.7%) reported at least one adverse effect during isotretinoin treatment, while 42 patients (9.3%) reported no adverse effects (Table 1). The most commonly reported adverse event was back pain in 60 patients (13.3%), followed by skin dryness in 57 patients (12.7%), muscle pain in 56 patients (12.4%), and constipation in 54 patients (12.0%). Hair loss was reported by 50 patients (11.1%), eye dryness by 46 patients (10.2%), lip dryness by 43 patients (9.6%), and nosebleeds by 42 patients (9.3%).

## 6 Discussion

Acne vulgaris is a chronic inflammatory skin disease in which systemic inflammation contributes to its progression [1]. Isotretinoin is the most effective treatment for severe cases, but its effects on hematological markers and systemic inflammation are not fully understood [2]. In our study, isotretinoin was initiated at a median age of 29 years, despite acne onset at 16 years, with a median disease duration of 9 years. This delay highlights the chronic nature of acne and the need for earlier intervention to prevent complications such as scarring and psychosocial distress. Previous studies have identified concerns about the side effects of isotretinoin, patient hesitation, and clinician reluctance as common reasons for delayed systemic treatment [3, 17].

The median BMI in our study was 26.30 kg/m^2^ (IQR: 22.40–30.33), placing most patients in the overweight range. Although some studies have linked higher BMI to acne through insulin resistance and hormonal imbalance, others have reported no significant association with prevalence [18, 19]. Our findings do not directly correlate BMI with isotretinoin response, but the potential role of obesity‐related inflammation in acne pathophysiology warrants further investigation.

Conflicting results have been reported regarding the effect of isotretinoin on inflammatory markers derived from CBC parameters. Our results show a significant reduction in systemic inflammatory markers, with a decrease in WBC and neutrophils and an increase in lymphocytes. Monocyte and platelet counts also showed significant reductions over 24 weeks. In addition, the significant decrease in NLR and MLR observed in our study suggests that isotretinoin down‐regulates systemic inflammation beyond its sebostatic effect. While some studies have reported a decrease in NLR levels following isotretinoin treatment [4, 5, 6, 7], similar to our findings, others have found no significant change [8, 9]. Previous reports suggest that isotretinoin inhibits neutrophil and monocyte chemotaxis by reducing TLR2 expression [10]. In addition, Karadag et al. observed a decrease in inflammatory cytokines such as TNF‐α, IL‐4, IL‐17, and IFN‐γ after treatment, supporting its role in suppressing systemic inflammation [11].

A novel aspect of our study is the evaluation of RDW/lymphocyte and RDW/platelet ratios as inflammatory biomarkers. RDW is increasingly recognized as a systemic inflammatory marker, with elevated levels reported in Behçet's and Crohn's disease, reflecting underlying inflammatory activity and oxidative stress. In our study, RDW, RDW/lymphocyte, and RDW/platelet ratios were significantly reduced during isotretinoin treatment, supporting its potential anti‐inflammatory effect. However, previous studies have reported conflicting results regarding the effect of isotretinoin on RDW. While some have observed an increase, suggesting a possible inflammatory response, others have found a significant decrease, attributing this to the immunomodulatory properties of isotretinoin [12, 13, 14, 15].

Interestingly, ALT, AST, and ALP levels did not change significantly throughout the study, supporting recent findings that routine liver enzyme monitoring in all isotretinoin‐treated patients may not be necessary unless risk factors such as pre‐existing liver disease or concomitant hepatotoxic medications are present [16].

Clinically, our study confirmed a significant reduction in acne lesion counts (comedones, papules, and pustules) and GAGS scores, consistent with the known efficacy of isotretinoin. The improvement in DLQI scores highlights the positive impact of treatment on patients' quality of life, an important but often overlooked aspect of acne management. In addition, the increase in SF‐36 physical and mental health scores and the decrease in HADS scores suggest that isotretinoin not only improves skin appearance but also alleviates psychological distress. Given the association between acne and depression, these findings further emphasize the importance of early and effective treatment to prevent deterioration in mental health [20].

Regarding adverse events, 90.7% of patients experienced at least one adverse event, which is consistent with the literature reporting that most isotretinoin users develop mucocutaneous symptoms. Musculoskeletal symptoms, constipation, and alopecia were reported more frequently in our cohort than in previous studies. Back pain (13.3%) and muscle pain (12.4%) exceeded the commonly reported range of 10%–20%, whereas constipation (12.0%) was higher than typically highlighted in isotretinoin safety profiles. Hair loss (11.1%) was also slightly more common than the 5%–10% reported in previous studies, suggesting potential variability based on dosage, treatment duration, or individual susceptibility [2, 21].

Our study has several strengths, including its large sample size, comprehensive analysis of inflammatory markers, and extended follow‐up. However, limitations include its retrospective design and the lack of a control group.

In conclusion, our study confirms the anti‐inflammatory effects of isotretinoin, as demonstrated by reductions in WBC, neutrophils, NLR, and MLR, together with significant improvements in acne severity, quality of life, and mental health. The identification of RDW/lymphocyte and RDW/platelet ratios as inflammatory markers introduces novel indicators of the systemic immunomodulatory effects of isotretinoin. Further research is needed to investigate the long‐term effects of isotretinoin on systemic inflammation and to confirm the clinical utility of RDW‐derived ratios as biomarkers.

## Ethics Statement

The study received approval from the institutional ethics committee (Decision Number: 2020/499). Written informed consent was obtained from all participants prior to study enrollment.

## Conflicts of Interest

The authors declare no conflicts of interest.

## Data Availability

The data that support the findings of this study are available from the corresponding author upon reasonable request.
